# Supplementary material for: Developing a xenograft model of human vasculature in the mouse ear pinna
Source: Sci Rep. 2020 Feb 6;10:2058. doi: 10.1038/s41598-020-58650-y (PMC7004987; doi:10.1038/s41598-020-58650-y)
Supplement: Supplementary file 2 — Supplementary Figures. [file 41598_2020_58650_MOESM2_ESM.pdf]

Developing a xenograft model of human vasculature in the mouse ear pinna

Gavin R. Meehan, Hannah E. Scales, Rowland Osii, Mariana De Niz, Jennifer C. Lawton, Matthias Marti, Paul Garside, Alister Craig & James M. Brewer

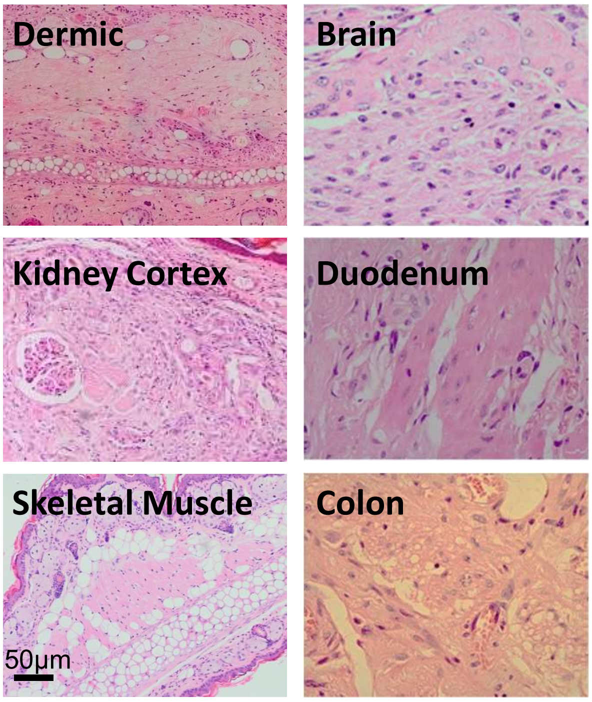

Supplementary Figure 1 – Alternative tissues can be implanted into the ear pinna.

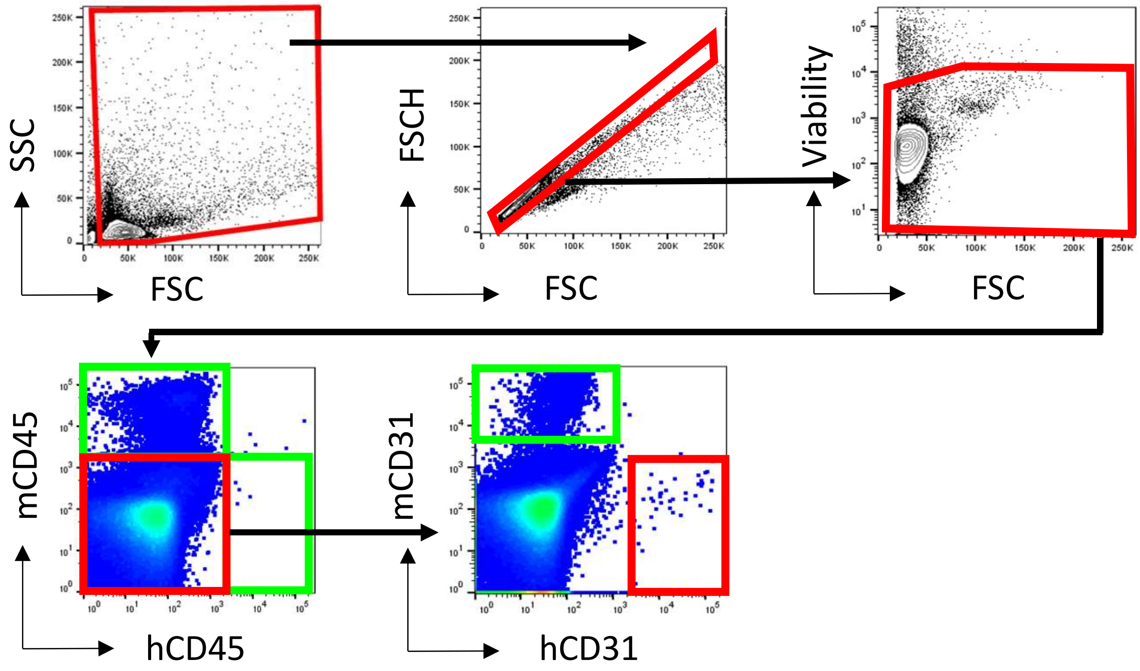

Supplementary Figure 2 – Gating strategy for identifying endothelial cells by flow cytometry

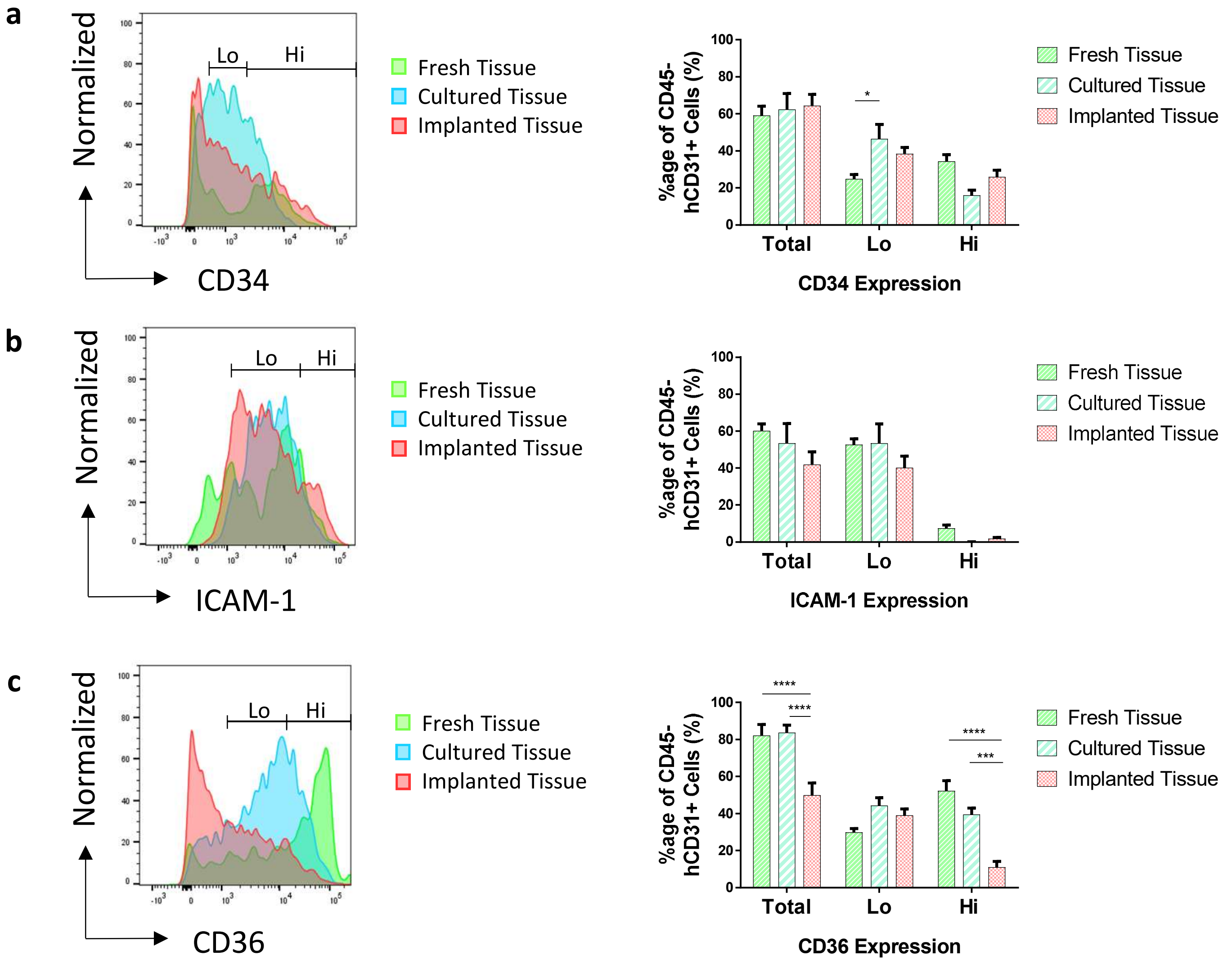

Supplementary Figure 3 - Changes in expression of endothelial cells markers

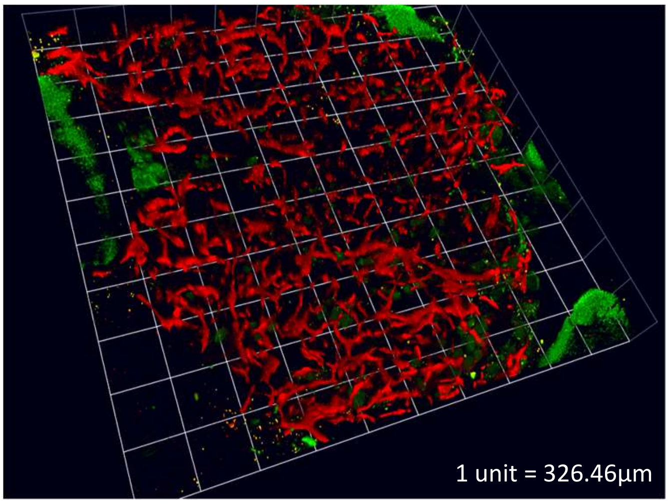

Supplementary Figure 4 – 3D Image of engrafted adipose tissue

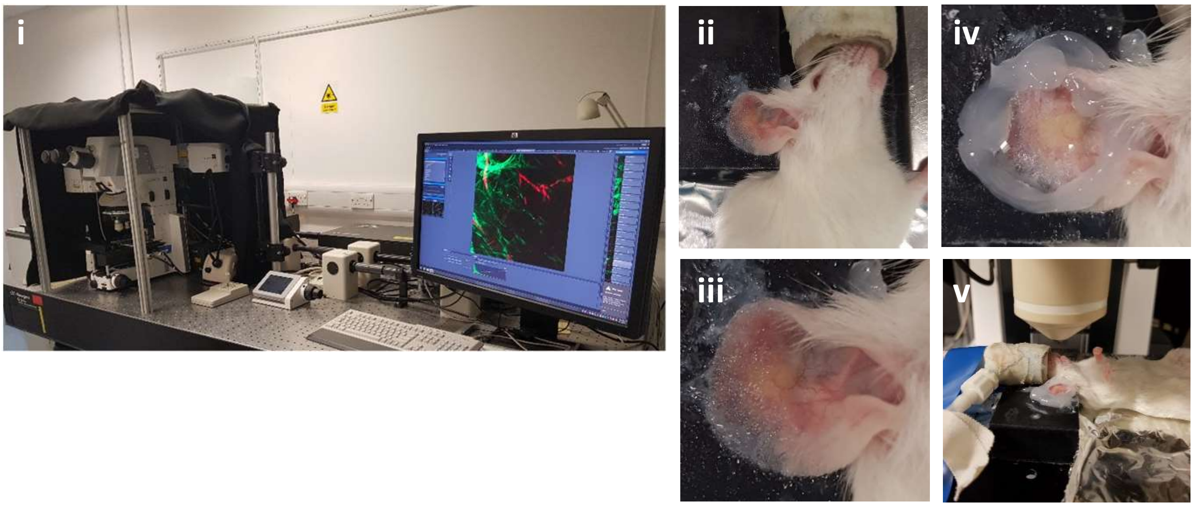

Supplementary Figure 5 – Intravital imaging of engrafted mice

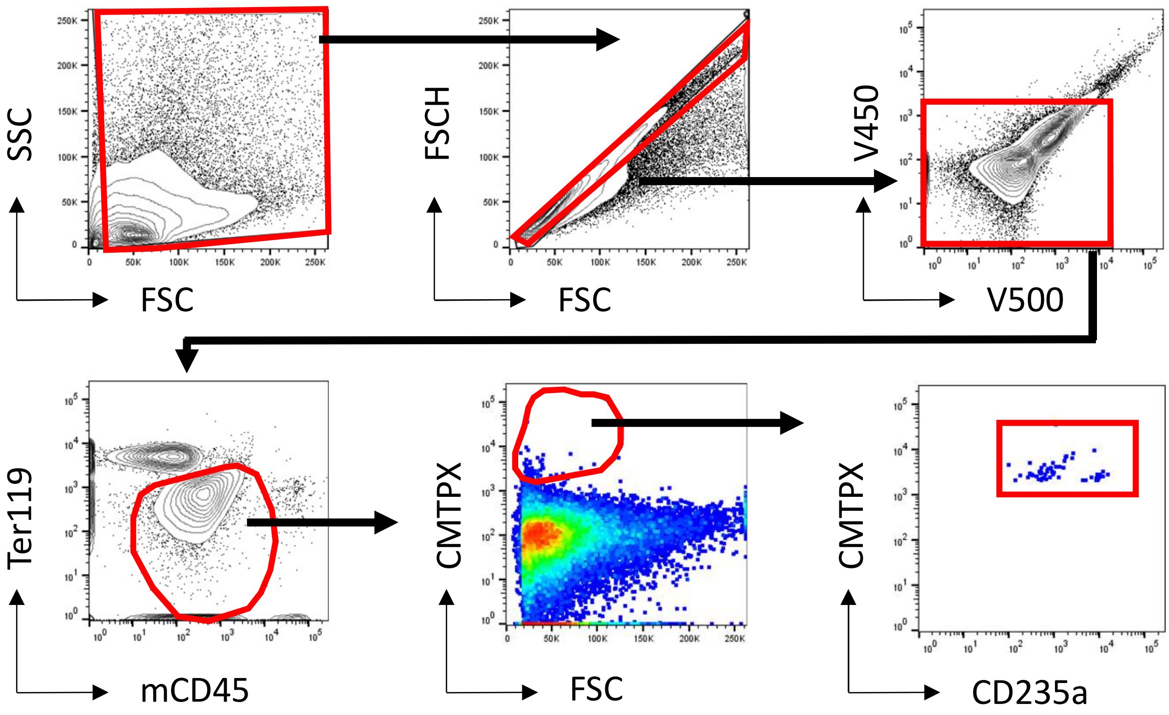

Supplementary Figure 6 – Gating strategy for identifying labelled human RBCs in mouse tissue
